# Prevalence and characteristics of unipolar mania in a low-income country setting: population-based data from the Butajira cohort, rural Ethiopia

**DOI:** 10.1007/s00127-022-02399-4

**Published:** 2022-12-24

**Authors:** Fredrik Wikström, Hugo Zeberg, Rosie Mayston, Lena Backlund, Abebaw Fekadu

**Affiliations:** 1 Psychiatry Southwest, Karolinska University Hospital Huddinge, Stockholm, Sweden; 2 Department of Population Health, London School of Hygiene and Tropical Medicine, Centre for Global Mental Health, London, UK; 3 Inst. for Molecular Medicine and Surgery, Stockholm and Centre for Molecular Medicine, Karolinska Institutet, Karolinska University Hospital, L8:00, S-171 76, Stockholm, Sweden; 4 Department of Neuroscience, Karolinska Institutet, Stockholm, Sweden; 5 Department of Global Health & Social Medicine, King’s Global Health Institute, NE Wing, King’s College London, London, UK; 6 Centre for Psychiatric Research and Health Care, Stockholm, Sweden; 7 Centre for Innovative Drug Development and Therapeutic Trials for Africa (CDT-Africa), Addis Ababa University, Addis Ababa, Ethiopia; 8 Department of Global Health and Infection, Brighton and Sussex Medical School, Brighton, UK; 9 Department of Psychiatry, College of Health Sciences, Addis Ababa University, Addis Ababa, Ethiopia; 10 Affective Disorders Research Group, Department of Psychological Medicine, Institute of Psychiatry, Psychology and Neuroscience, King’s College London, London, England

**Keywords:** Unipolar mania, Bipolar disorder, Ethiopia, Low-income country

## Abstract

**Purpose:**

Previous research suggests unipolar mania, i.e., bipolar disorder without depression, to be more common in low-income countries. However, longitudinal population-based studies on unipolar mania from low-income countries are lacking. This study therefore examined unipolar mania, in Butajira, Ethiopia, and associations with possible determinants.

**Methods:**

Key informants and 68,378 screenings with the Composite International Diagnostic Interviews (CIDI 2.1) identified suspected cases of bipolar disorder. Diagnosis was confirmed using the Schedules for Clinical Assessment in Neuropsychiatry (SCAN 2.1) (*n* = 2,285). 315 participants with bipolar disorder were recruited and followed up for an average of 2.5 years. Unipolar mania was defined when illness episodes consisted of at least two manic relapses. 240 cases had sufficient data to ascertain course of disorder.

**Results:**

41.7% (100 of 240 cases) of participants had unipolar mania. Unipolar mania was associated with less suicidal ideation (0% vs. 26.4%, *p* < 0.001), less suicidal thoughts (occasionally/often: 1%/3% vs. 19.6%/21%, *p* < 0.001), and less history of suicide attempt (2% vs. 11.6%, *p* = 0.01). The participants with unipolar mania tended to have better social functioning (OR = 2.05, *p* = 0.07) and less alcohol use (20.8% vs. 31.4%, *p* = 0.07). The study was partly based on retrospective data liable to recall bias. Some cases defined as unipolar mania in our study may later develop depression.

**Conclusion:**

Previous cross-sectional studies finding high proportions of unipolar mania in low-income countries appear supported. Unipolar mania trended towards better social functioning and was associated with lower suicidality. Future unipolar mania specifications could inform treatment and prognostic estimates of bipolar disorder.

**Supplementary Information:**

The online version contains supplementary material available at 10.1007/s00127-022-02399-4.

## Background

Bipolar disorder is a chronic and severe disorder, with alternating and mixed states of depression and mania, normally with relatively symptom free intervals in between. Depression is often characterised by low mood and loss of interest, decreased energy, and negative thoughts including suicidal ideation. Mania can be seen as a counterpart, typically with an expansive state of mind, elevated mood, increased activity, energy, and impulsivity, but with a decreased need for sleep, and behaviours which can have far reaching negative consequences. Delusions and psychoses can be symptoms of both severe depression and mania. The suicide rate of bipolar disorder has been estimated at 6–15% [1]. The global prevalence is around 1% [2], and bipolar disorder accounts for 0.5% of the global burden of disease, and 7% of the burden associated with mental and substance use disorders [3, 4].

Some patients seem only to experience episodes of mania and never depression, i.e., only one side of the polarity. However, in today’s classificatory systems, The Diagnostic and Statistical Manual of Mental Disorders 5 (DSM-5) and The International Statistical Classification of Diseases and Related Health Problems 11 (ICD-11), the only available diagnosis for this clinical picture is bipolar disorder. The systems differ slightly; DSM-5 labels this bipolar disorder, while ICD-11 has the distinction “other bipolar affective disorders”, with the sub-label “recurrent manic episodes”. The previous versions DSM-IV and ICD-10, which were used during the recruitment for the current study, are similar in this regard [5, 6].

An increasing number of scientific papers, however, argue that unipolar mania (UM), i.e., mania without depression, as opposed to bipolar disorder with both depression and mania (DM), warrants a separate diagnosis. The most recent comprehensive reviews argue strongly that UM is substantially different from DM and should be seen as an entity of its own [7–9]. A study by Merikangas [10] suggests a possible independent familial transmission of mania and depression, thus questioning the bipolar unity, while a recent study using UK biobank data found “key differences” between UM and DM [11]. Yet, other papers conclude that UM is “clinically distinct” [12] and “a clinical reality in our daily practice” [13].

There is some evidence that the presentation of bipolar disorder may differ by region. For example, there may be a lower age of onset, less depression, and more mania in countries nearer the equator [14, 15]. In high-income countries (HICs), results from three prospective studies ranged between 8.6 and 15.8% (mean = 11%) prevalence of UM (of those diagnosed with bipolar disorder), while seven retrospective studies ranged between 11.0 and 37.7% (mean = 23.1%). In 13 retrospective studies from low- and middle-income countries (LMICs), the proportion of UM varied between 5.4 and 65.3% (mean = 35.9%) [8]. However, there is a lack of longitudinal population-based studies examining the prevalence and course of UM in LMICs.

The definition of UM remains debated, but there is a call for consensus regarding needed number of manic episodes and follow-up time [9]. One review recommends using the definition of at least two manic episodes as “possible UM” and at least four manic episodes as “probable UM” [16]. It is not, however, possible to define UM with full accuracy. Research participants thought only to experience mania might later go on to develop depression beyond the lifetime of the study. Including follow-up data can mitigate this problem, but one would have to follow the entire life of an individual to fully ascertain the definition. Thus, diagnostic stability of UM, meaning not reclassifying due to occurring depression, varies between studies. For example, a study requiring one episode of mania for the UM definition had a stability of 75% over 10 years, whereas another study using the same threshold had a stability of 26% over 15–20 years [8].

Previous studies have found associations between UM and various clinical features. In studies from HICs, UM in bipolar disorder is associated with an earlier age of onset, hyperthymic temperament, and higher cardiovascular mortality [8]. Other findings are better social adjustment and more use of stimulants such as amphetamine and marijuana [17], as well as less suicidality [6, 7]. A recent international study pooling data from nine studies also found UM to have less comorbid substance use disorders and a male preponderance in UM [7]. Studies on UM in LMICs are scarce. Two Turkish studies looking at UM have found a lower age of onset, less suicidality, less rapid cycling, and to be less responsive to lithium treatment compared to DM [18] as well as worse visual memory and executive functions [19]. A recent Indian multi-center study identified lower suicidality and higher concentrations of lithium in UM [20]. Two African studies on UM have found a higher proportion of UM in males [21, 22].

If confirmed, transcultural differences in presentation of bipolar disorder may have implications for the treatment of psychiatric disorders. For example, the absence of the first-line treatment lithium in LMICs [23] may be less problematic for UM, since UM may respond less well to this treatment [18]. Previous research has also suggested the exploration of alternative pharmacological treatment models in LMICs [24]. Differing presentations may also give clues to the biological and psychosocial aetiology of bipolar disorder and UM.

The aim of the present study was to investigate the prevalence of UM in a cohort of people diagnosed with bipolar disorder in a low-income country (Ethiopia), and to explore possibly associated features. Four variables where previous evidence suggests possible associations with UM were analysed for validation.

We have not identified any previous true prospective or population-based studies on UM from low-income countries [8]. This cohort is also unique in that it was largely treatment naïve at recruitment (93%) [24], which may give a better insight into the natural presentation of UM.

## Methods

### Setting

Ethiopia is a predominantly rural low-income country in Eastern Africa situated near the equator, with a population of around 110 million. The only specialist psychiatric hospital is situated in the capital and has around 280 in-patient beds. Psychiatrists number about 70 with numbers continuously growing very slowly through two training programs. Various cadres of middle- and low-level mental health practitioners also provide care. However, the vast majority of people living with bipolar disorder do not receive psychiatric care [25].

Data collection for this study took place in the Butajira district, which is a demographic surveillance site established in 1986. A census estimated the total population to be 227,135 in 1994 [26]. It has been the research platform for a large number of studies examining different health-related problems [27]. A cohort of patients with bipolar disorder has been followed up since the inclusion to the Butajira mental health research project in 1997–2001 [24, 28–30].

The stimulant drug khat is common in Ethiopia and consumed by around 50% of the population in the southern part of the country. Khat is commonly thought to increase energy and concentration and to alleviate stress [31]. Prolonged use is strongly associated with elevated psychiatric symptoms, including depression and psychosis. There is a lack of prospective and controlled studies, however, and a causative link between khat and psychiatric disorders has not been proven [32].

The Butajira area is predominantly rural and health care services are limited. Prior to the project, there was no psychiatric service in the district. The project, however, established a mental health service in Butajira [24, 28].

### Study design

**Fig. 1:**
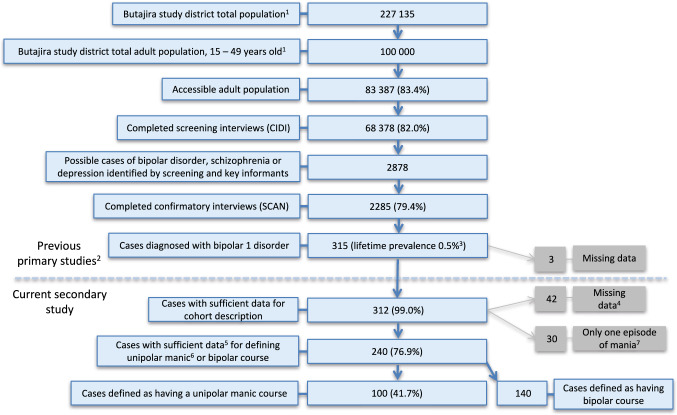
Defining unipolar manic and bipolar course, cohort selection, Butajira, Ethiopia. Percentages refer to preceding sum. 1 Census prior to data collection, 1994. 2 Baseline data collected 1997–2001. Follow-up data collected for an average of 2.5 years. 3 [24] 4. Missing baseline or follow-up data needed for defining course. 5 Including follow-up data. 6 Two episodes of mania or more. 7 Cases not possible to define as having either a unipolar manic or a bipolar course

The initial cross-sectional door-to-door survey in 1997–2001 screened the whole Butajira district population, aged 15–49, for bipolar disorder, schizophrenia, and major depression, with the CIDI interview administered to 68,378 individuals. The age limit of 49 was chosen for efficiency, because life expectancy was reported to be 50 years [33]. From this, 2,878 potential cases were identified and 79.4% completed confirmatory SCAN interviews. 315 patients were diagnosed as having bipolar I disorder, of whom 312 were available for this report [24, 28, 30]. Participants were eligible for inclusion if they and had lived in the area for at least 6 months. Exclusion criteria were bipolar II disorder and organic manic disorder after assessment by a physician. Of the available cases with bipolar disorder, 30 participants were excluded, because they only had a single episode and 42 were excluded because of missing data. Thus, a total of 240 participants were included in our analysis (Fig. 1).

### Instruments

The retrospective data used for the current report were collected during the baseline survey 1997–2001. The prospective follow-up data in the study were collected for an average of 2.5 years (range 1–4) and only concern number and type of illness episodes.

Initial screening for bipolar disorder and other severe mental disorders used the Composite International Diagnostic Interview 2.1 (CIDI 2.1) [34] and trained key informants to identify people with severe mental illness [35]. Those suspected to have bipolar disorder from the CIDI interviews or key informant data were assessed for confirmation of diagnosis with the Schedules for Clinical Assessment in Neuropsychiatry 2.1 (SCAN 2.1) [28, 36, 37]. Various instruments were also used to establish the severity and course of illness including annual follow-up data on affective episodes [Additional file 1]. The Short form Health Questionnaire (SF-36) has been validated for use to measure functioning and health-related quality of life [38], and for use with bipolar disorder [39]. The instrument has eight subscales and the social functioning sub-scale with two items measures the effect of physical and emotional health on normal social activities [28, 40].

### Main outcome: UM

The definition of UM was not pre-specified. For maximum validity, the aim was to use the highest number of manic episodes possible, guided by the recommendations by Harish et al [16], without compromising statistical analysis, due to the exclusion of cases. Diagnostic stability of UM for each of the definitions at baseline was calculated as the percentage of UM cases not experiencing a depressive or mixed episode during follow-up.

Six increasingly strict definitions of UM were explored, requiring one, two, or three episodes of mania, and including or excluding follow-up data [Additional file 1]. Cohort size decreased with stricter definitions due to the exclusion of cases with less manic episodes than needed to meet the definition. The resulting number of included cases ranged from 289 (UM definition = one manic episode, not including follow-up data) to 187 (UM definition = three manic episodes, including follow-up data). At least two episodes of mania without any depressive episodes at any stage of the follow-up was chosen as UM definition. This choice was made to meet the definition by Harish et al [16] of probable UM and because even stricter definitions would lead to the exclusion of more than 40% of cases which would compromise further analysis.

### Data analysis

Data analyses were carried out using Stata 13.1. The associations between subgroup classification UM or DM and potential confounders as well as correlates were calculated using chi-squared tests and Fisher's exact test when appropriate (Table 1).

**Table 1:** Demographic and clinical characteristics of unipolar and bipolar subgroups

Variable	Unipolar manic course (*n* = 100)		Bipolar course (*n* = 140)		*P* value^1^
	(*n*)	(%)	(*n*)	(%)	
Sex (*n* = 240)					
Male	59	59,0	72	51,4	0.25
Female	41	41,0	68	48,6	
Age in years (*n* = 240)					
15–19	14	14,0	13	9,3	0.39
20–29	39	39,0	56	40,0	
30–39	34	34,0	43	30,7	
40–49	13	13,0	28	20,0	
Mean (SD)^2^	28.8	8.01	29.9	8.51	0.31
Residence (*n* = 227)					
Urban	19	20,9	23	16,9	0.45
Rural	72	79,1	113	83,1	
Education level (*n* = 208)^3^					
Not literate	55	62,5	76	63,3	0.69
Elementary	21	23,9	32	26,7	
Secondary	12	13,6	12	10,0	
Occupation (*n* = 235)					
Not working	44	44,4	59	43,4	0.20
Paid work	24	24,2	22	16,2	
Domestic work	31	31,3	55	40,4	
Marital status (*n* = 240)					
Single/never married	32	32,0	37	26,4	0.63
Married	62	62,0	93	66,4	
Other	6	6,0	10	7,1	
Number of children (*n* = 227)					
0	30	33,0	45	33,1	1.00
1–3	28	30,8	42	30,9	
4 or more	33	36,3	49	36,0	
Religion (*n* = 225)					
Muslim	75	77,3	99	77,3	1.00
Christian	22	22,7	29	22,7	
Family history of mental illness (*n* = 232)					
No	79	79,8	108	81,2	0.79
Yes	20	20,2	25	18,8	
Alcohol experience (*n* = 233)					
Never	76	79,2	94	68,6	0.07
Once or more	20	20,8	43	31,4	
Khat use^4^ (*n* = 236)					
No	94	96,9	126	90,6	0.13
Mildly distressing	1	1,0	9	6,5	
Moderately distressing	2	2,1	4	2,9	
Tobacco experience (*n* = 188)^3^					
Never	75	96,2	99	90,0	0.16
Once or more	3	3,8	11	10,0	
Age of onset of disorder in years (*n* = 231)					
15 or under	13	13,5	23	17,0	0.89
16–20	38	39,6	49	36,3	
21–25	23	24,0	33	24,4	
26 or above	22	22,9	30	22,2	
Mean (SD)^2^	21.8	6.84	21.7	7.37	0.95
Duration of illness in years (*n* = 240)					
Mean (SD)^2^	7.62	8,34	9.30	8.34	0.10
Social functioning^5^ (*n* = 226)					
Above population mean	19	20.9	21	15.6	0.30
Mean (SD)^2,6^	62.9	25.31	57.5	26.15	0.12
Suicidal ideation (*n* = 240)					
No	100	100,0	103	73,6	** < 0.001**
Yes	0	0,0	37	26,4	
Thoughts about suicide (*n* = 238)					
Not at all	96	96,0	82	59,4	** < 0.001**
Occasionally	1	1,0	27	19,6	
Often	3	3,0	29	21,0	
History of suicide attempt (*n* = 238)					
No	98	98,0	122	88,4	**0.01**
Yes	2	2,0	16	11,6	

Categories from the original data-set recoded for analytical purposes; for details, see: Additional file 1

^1^Chi-squared test. Fisher’s exact test for cross-tabulations where number < 5. *T* test for means

^2^Data possibly not normally distributed. For median and IQR, see: Additional file 1

^3^Missing data for more than 10% of the cases (tobacco experience: 22.0% in UM and 21.4% in DM, and education level: 12% in UM and 14.3% in DM)

^4^Khat use by the proxy variable “problems due to alcohol or other substances”

^5^SF-36 Social functioning sub-scale. Range 0–100. Higher score indicates better functioning

^6^General population mean = 91.2 (SD 17.2) [26].

Crude odds ratios for correlates were calculated in four models using logistic regression and exact logistic regression in one model with a small number in one of the co-variates. Akaike Information Criterion (AIC) was used to fit the best model controlling for potential confounders. Lower AIC score per number of observations indicate a better fit [41]. AIC scores of models including age, gender, and all possible combinations of ten additional potential confounders were calculated. The model with the best fit included variables age, gender, alcohol use, occupation, social functioning, and age of onset with an AIC/observation score of 1.349. This model was used for the calculation of all adjusted ORs (Table 2). Histograms indicated a non-normal distribution of age of onset and the variable was recoded to interquartile range (IQR) categories for the modelling.

**Table 2:** Relationship between unipolar mania and gender, khat use, social functioning, and age of onset

Variable	*n*	OR (95% CI)	*p *value	Adjusted OR^1^	*p *value
Model A					
Gender (male)	240	1.36 (0.81–2.28)	0.25	1.54 (0.62–3.82)	0.35
Model B					
Khat use^2^	236	0.31 (0.06–1.18)	0.07^3^	–	–
Model C					
Social functioning^4^	226	1.43 (0.72–2.85)	0.31	2.05 (0.93–4.48)	0.07
Model D					
Age of onset^5^ (0–17, 18–20, 21–25, 26–49)	231	1.19 (0.58–2.47)	0.64	1.48 (0.62–3.53)	0.38
		1.06 (0.52–2.15)	0.88	1.41 (0.56–3.50)	0.46
		1.11 (0.54–2.29)	0.77	1.42 (0.51–3.92)	0.50

Logistic regression with bivariate models and a multivariate model controlling for potential confounders. Optimal adjusted model was selected comparing Akaike Information Criterion (AIC) scores (see Methods section). Model B was not further analysed due to extreme mismatch between reported use and previously shown prevalence

^1^*N* = 205 in adjusted model

^2^Khat use by the proxy variable “problems due to alcohol or other substances”. Categories 2–3 (see Table 1) collapsed to increase number in cell

^3^Exact logistic regression due to one cell of “khat use” containing only three observations

^4^SF-36 social function sub-scale, score above population mean

^5^Categories by median and IQR

## Results

The proportion with UM was 41.7% (*n* = 100/240). Of the 110 with UM at baseline, 24 developed depression during the 2.5 years of follow-up, yielding an estimated diagnostic stability of 76.4%.

### Characteristics of unipolar manic and bipolar subgroups

Clinical and demographic characteristics of the UM and DM subgroups can be seen in Table 1. Mean age for UM and DM were similar with 28.8 and 29.9 years, respectively. The duration of illness varied slightly between the groups with a mean of 7.62 years for UM and 9.30 years for DM.

A clear difference was observed in reported suicidality between UM and DM, with less suicidal ideation (0% vs. 26.4%, *p* < 0.001), less suicidal thoughts (occasionally/often: 1%/3% vs. 19.6%/21%, *p* < 0.001), and less history of suicide attempt (2% vs. 11.6%, *p* = 0.01). Another notable difference was alcohol experience (never vs. once or more) with 20.8% of the UM group having tried once or more versus 31.4% in the DM group (*p* = 0.07). In the post hoc exploration of the remaining SF-36 subscales, 13 out of 14 measures indicated better functioning for UM [Additional file 1].

### Correlates, bivariate and multivariate logistic regression

Few of the investigated variables showed any association with UM. Social functioning showed a trend towards significant association with UM (OR = 2.05, 95% CI = 0.93–4.48, *p* = 0.07); in a model with six co-variates in addition to social functioning. Khat use similarly trended towards significance (OR = 0.31; *p* = 0.07; as a single regressor). Multivariate logistic regression of khat use was aborted due to the unexpected very low consumption and an extreme mismatch between reported use and expected use based on previous studies. See Table 2 for description of regression models.

## Discussion

This prospective study examined the prevalence of UM in a low-income country setting consisting of a large bipolar cohort in Butajira, Ethiopia. The main finding was a relatively high rate of UM, amounting to 41.7% of the bipolar cohort. Our finding is consistent with the limited available evidence from LMICs. The rate of UM has generally been found to be much lower in HICs, with a pooled average of available studies estimated to 19.1% and an average of 11.0% if only including the three available prospective studies (follow-up ranging 3–26 years) as reported by Angst and Grobler (2015).

Underlying reasons for the high proportion of UM in LMICs are unclear, partially because the exact aetiology of bipolar disorder and UM remains unknown. Recall bias may be an important inflating factor. The heritability of bipolar disorder is above 50% [42], which, together with the differing hereditary traits of UM [8], hints that genetic factors may be important. The socioeconomic and cultural differences between LMICs and HICs may also affect the clinical presentation of mental disorders. However, studies have so far failed to outline any important sociodemographic differences between DM and UM. An interesting hypothesis is that proximity to the equator, which affects sunlight variation and circadian rhythm (believed to be an important factor of bipolar disorder), might play a part [14]. The clinical presentation may also differ comparing to HIC settings due to the fact that only about 7% of bipolar cases were receiving treatment in the baseline study [30].

This study contributes to the debate regarding the UM construct, supporting this construct by demonstrating a trend towards an association with better social functioning as well as associations with lower suicidality. A recent study pooling nine cohorts supports the validity and distinct quality of UM suggesting importance for clinical care and further research [7]. In our study, we found no significant associations between UM and the analysed correlates of UM, namely gender, social functioning, khat use, and age of onset. One explanation for this may be that several variables demonstrated wide confidence intervals, suggesting that the study was not sufficiently powered to either detect or rule out associations. The previously shown high prevalence of the psychostimulant khat in the area (50%) [31] was unexpectedly not matched by this study (7%) and further analysis and interpretation of this possible determinant must be seen as unreliable. Hypothetical explanations for this mismatch include measurement error as well as decreased intake after the psychiatric evaluation and clinical diagnosis. A recent meta-analysis found strong associations between khat use and depression (OR = 2.39) as well as psychosis (OR = 1.47) [32]. If the link were to be causative, khat use could indeed affect the classification of DM/UM. Prospective and controlled studies to clarify this relationship are needed.

The association between UM and better social functioning in the adjusted model C (*p* = 0.07), together with post hoc findings that the remaining 13 out of 14 SF-36 sub-scale measures were higher in UM (four differences with a significance level below *p* = 0.05), supports earlier results of a better social functioning in UM. Replicating a high prevalence and indications of better social functioning may be seen as support for the construct validity of UM. One reason for a better social functioning could be that mania usually has shorter episode duration with a higher rate of remission than depression [43]. Mania may also constitute more evident malady and thereby more frequently lead to help and treatment, exemplified by a recent small study in France which found a higher rate of psychiatric admissions in UM [44]. Along with the finding of low suicidality in UM compared with DM, the higher social functioning scores can be seen as indicative of a lower mental health burden in UM compared with DM. The well-known link between depression and suicide may explain part of the lower suicidality in UM [45].

Given previous findings indicating a different symptomatology and also treatment response, e.g., to lithium for UM as compared to DM [18] these findings could have implications for the care and treatment of bipolar disorder in a country such as Ethiopia.

However, further studies are needed to verify the distinct qualities of UM before direct clinical considerations can be made.

### Strengths and limitations

There are no prior studies on UM in LMICs which used a population-based study design. Another strength of this study is that findings emanating from this data-set are potentially generalizable to other regions of Ethiopia, and may be relevant to other rural populations across sub-Saharan Africa [24]. The use of follow-up data is crucial for the study of UM, reducing the effect of recall bias, and enabling an estimation of the diagnostic stability of UM. The fact that the cohort was largely treatment naive (93%) [24] might also give insights into the natural presentation of UM. Most instruments had also been validated in the local setting and were administered by trained clinicians, nurses, or high school graduates increasing quality of collected data [Additional file 1].

The definition of UM in this study required two episodes of mania. The higher the number of experienced manic episodes, the more stable the definition of UM, which would increase validity of findings. Fewer cases will be redefined as having a bipolar course because of later experiencing depression. Using a stricter definition was not possible, however, because of the resulting exclusion of cases not meeting definition (i.e., too few episodes of mania). As can be seen in Fig. 1, around one-fifth of individuals were lost in three different steps of the previous study which may lead to selection bias. Another limitation of this study is that the UM definition of two manic episodes led to the exclusion of individuals with only one manic episode. The retrospective design concerning previous episodes may lead to recall bias with mania being more easily remembered. Another important aspect is that the CIDI might have a low sensitivity for detecting milder forms of depression [46–48] as well as bipolar disorder [49] in Ethiopian settings. This would inflate the number of manic episodes along with the proportion suffering from a unipolar manic course. The relatively short duration of follow-up in our study together with the retrospective nature of assessment may also have contributed to the high proportion of UM identified.

## Conclusion

In this the first ever population-based prospective study on UM in a LMIC, we present supportive evidence of the previous findings that the UM proportion of bipolar disorder is higher in LMICs. Results also indicate a possibly better social functioning and there was a significant and markedly lower suicidality in the UM group on all three measures.

Further validation of the UM construct is warranted with more prospective long-time follow-up studies of UM. This would decrease the potential effects of recall bias and misclassification. Additional collected unpublished follow-up data from the Butajira cohort could be analysed to complement this study with a longer follow-up increasing the validity of the UM definition and related findings.

## Supplementary Information

Below is the link to the electronic supplementary material.

Supplementary file1 (DOCX 42 KB)

## Data Availability

The data that support the findings of this study are available from AF, but restrictions apply to the availability of these data, which were used under license for the current study, and so are not publicly available. Data are, however, available from the authors upon reasonable request and with permission of AF.

## Abbreviations

AIC: Akaike information criterion
DM: Bipolar disorder with both depression and mania
HIC: High-income country
IQR: Interquartile range
LMIC: Low- and middle-income country
SD: Standard deviation
UM: Unipolar mania
