# Supplementary material for: Prevalence and characteristics of unipolar mania in a low-income country setting: population-based data from the Butajira cohort, rural Ethiopia
Source: Soc Psychiatry Psychiatr Epidemiol. 2022 Dec 24;58(4):537–45. doi: 10.1007/s00127-022-02399-4 (PMC10066167; doi:10.1007/s00127-022-02399-4)
Supplement: Supplementary file 1 — Supplementary file1 (DOCX 42 KB) [file 127_2022_2399_MOESM1_ESM.docx]

## Additional file 1

Additional file 1 includes additional information regarding study setting, instruments used, subject identification and design of original study, recoded variables, definition of affective, episodes, origins of secondary outcome data, results: IQR – interquartile ranges.

## Setting

This thesis is based on cross-sectional and limited follow-up epidemiological data from a study on schizophrenia and bipolar disorder, which was part of the Butajira rural health project (Kebede et al. 2003; 2006; Fekadu et al. 2006). The Butajira project was carried out in the Meskan and Mareko District in south central Ethiopia and established a demographic surveillance site, which ran continuous health and demographic surveillance for 10% of the population. The district capital town, which is located 135 km south of Addis Ababa, is named Butajira and the whole district is also commonly referred to as Butajira. The area is predominantly rural and situated at an altitude of 1500 to 3400 meters above sea level.

A census prior to the project was carried out in 1994 and estimated the total population to be 227,135. A majority of the population is from the Gurage ethnic group and of Islamic faith. Agriculture is the dominating trade. The local language is Guragigna but the national language Amharic is also widely spoken. Health care is limited and accessible only in towns and to some degree at health posts in sub-districts. Prior to the project there was no psychiatric service in the district. The project, however, made the establishment of a mental health service in the district possible (Kebede et al. 2006; Fekadu et al. 2006).

## Instruments

Initial screening used the CIDI 2.1 (Composite International Diagnostic Interview 2.1) (WHO 1997) and trained key informants to identify probable cases with severe mental illness. The combination of these two methods has been found effective (Shibre et al. 2002).

The CIDI 1.0 was initially translated into Amharic and evaluated (E Rashid, D Kebede, and A Alem 1996) and subsequently updated to version 2.1. Reliability and acceptability of the CIDI in Ethiopia have been found acceptable (E Rashid, D Kebede, and A Alem 1996). CIDI-cases were confirmed with DSM-IV diagnoses using the Amharic version of the SCAN 2.1 (Schedules for Clinical Assessment in Neuropsychiatry) (Fekadu et al. 2006; Wing et al. 1990; Andrews and Peters 1998). The SCAN was translated into Amharic by two psychiatry residents with formal WHO SCAN training and subsequently developed together with consultant psychiatrists. Satisfactory agreement between interviewers was reached in clinical and community settings but no formal reliability testing of the SCAN was done.

Correct use and validation of instruments are important to minimise the risk for classification error. As can be seen above, many steps have been taken to decrease this risk. Following is an excerpt from the PhD thesis (Fekadu 2010) laying the ground for the current study, further commenting on this risk: *“Classificatory error is an important consideration. But this seems unlikely given the care taken to ascertain the cases. First cases were identified through a clinical interview using the SCAN. Second, interviewers had access to second opinion when they were in doubt of the diagnosis of a disorder. Third, an additional diagnostic step was required when it was found that most of the identified cases had bipolar disorder. This third step involved clinical diagnosis by senior psychiatrists trained in the UK. In fact, this third step excluded cases who were categorised as having bipolar disorder and may have led to underestimation in the actual prevalence of bipolar disorder.”*

Baseline assessment also included the YMRS (Young Mania Rating Scale)(Young et al. 1978), the HDRS (Hamilton Depression Rating Scale) (Hamilton 1960), the SF-36 (Short-Form-36 Health Survey) (Ware 1993) (see below for description), the BISS (Basic Information on Study Subjects), and the LCSS (Life Chart Score Sheet) (Sartorius and Janca 1996). Annual follow-up data on affective episodes (manic, depressive, and mixed according to DSM-IV) was collected using SCAN, YMRS & HDRS. The BISS and SF-36 were administered by trained psychiatric nurses, the CIDI by high school graduates trained by WHO-affiliated trainers, and follow-up data collection as well as other baseline assessments were carried out by psychiatric residents, having formal SCAN training and at least 4 years of experience in clinical psychiatry. A data manager was employed full time controlling completeness, consistency, and accuracy of all questionnaires. Double data-entry was employed.

The SF-36 is widely used for measuring outcomes of mental illness and has been validated for measuring functioning and health related quality of life (Brazier et al. 1992), and for use with bipolar disorder (Leidy et al. 1998). The Amharic translation has been validated in Butajira (Kebede et al. 2004). The instrument includes 36 items yielding 8 subscales. A scoring algorithm authored by the developers was used to calculate sub-scale scores (Ware and Sherbourne 1993). The social functioning sub-scale with two items measures the active effect of physical and emotional health on normal social activities. The remaining seven subscales (physical functioning, role-physical, role-emotional, bodily pain, vitality, mental health, and general health perception) have been described in the original study. Scores of sub-scales range from 0 to 100 and higher SF-36 scores indicate better functioning (Kebede et al. 2006).

## Subject identification and design of original study

The original study was a cross-sectional door-to-door survey screening the whole population for bipolar disorder, schizophrenia, and major depression. Prisons were included. One of the 45 sub-districts, with an estimated 17,000 people in the targeted age range, was inaccessible for screening. Of the 68,378 CIDI interviews 42.5% were carried out with males and 57.5% with females. Of the subsequent 2878 potential cases, 719 had been identified by key informants, and 79.4% completed confirmatory SCAN interviews. 315 patients were diagnosed as having bipolar 1 disorder, 321 as having schizophrenia, and 212 as having major depression, according to DSM-IV criteria.

Single manic episode qualified as bipolar disorder. Annual follow-up data on affective episodes for an average of 2.5 years were available for the current study. Of 264 cases 30.3% had four yearly assessments, 38.6% had three, 19.3%% had two, and 11.7% had one yearly follow-up assessment (Kebede et al. 2006; Fekadu et al. 2006). Inclusion criteria were age 15-49 and having lived in the area for at least 6 months. Exclusion criteria were bipolar II disorder and organic manic disorder after physical and neurological assessment by a physician. The age limit of 49 was chosen for efficiency and the fact that life expectancy was reported to be 50 years (Byass et al. 2002).

### Recoded variables

Age was coded into decennial categories for practical and comparative reasons. Occupational status (SCAN item 0.027: Full time paid, Part time paid, Household duties, Unemployed, Retired, Disablement, Pensioneer, Sheltered work, Primarily a student) was recoded into three categories (Not working, Paid work, Domestic work). Marital status (SCAN item 0.024: Married, Cohabiting, Separated, Divorced, Widowed, Never married and Not cohabiting) was recoded into three categories (Single/never married, Married, Other). Number of children (0-20) was recoded into three categories (0, 1-3, 4 and more) and age of onset of disorder was recoded into 4 categories (0-15, 16-20, 21-25, 26-49), based on the author's subjective judgment of relevance. Suicidal ideation (HDRS item 3: Absent, Feels life is not worth living, Wishes he/she were dead or any thoughts of possible death to self, Ideas or gestures of suicide, Attempts at suicide) was recoded into 2 categories (No, Yes). For the logistic regression modelling the variable "khat use" was recoded to binary categories because of small numbers.

### Definition of affective episodes

Affective episodes used for the unipolar mania (UM) definitions were:

*(numbers indicate SCAN item number)*

a) number of previous affective episodes (1.053),

b) depression or mania at baseline (1.003 & 1.004. These were used by the original research team to indicate severity of any current episode. Mild, moderate, and severe distress were all recoded to current affective episode.).

c) in case of missing data on previous episodes, age of onset of manic disorder (1.017) were coded as a previous manic episode, if the age of onset differed from the current age by at least one year or if no manic episode was present at baseline.

d) three variables indicating any episode, any manic episode and any depressive episode during follow-up were provided by the holder of the original data. "Any manic episode" during follow-up was coded as one manic episode.

e) any registered age of onset of depression (1.016), any registered start date of a depressive episode (1.012), any depression at baseline (1.003), any depressive episode during follow-up, any "any episode" during follow-up not accounted for by manic or depressive episode during follow-up (indicating a mixed episode).

All cases with a depressive or mixed episode (e) were defined as having a bipolar course with both depression and mania (DM). Manic episodes (a-d) were summed for each case to make up the total number of manic episodes. Cases without a registered depressive episode at baseline, but where the data also failed to indicate a specific manic episode at baseline (due to missing data), were defined as having one single episode of mania, since all cases are diagnosed with bipolar disorder which required at least one manic episode. Cases were excluded for definitions requiring two episodes of mania if data indicated two episodes but had missing data on the specific episodes.

### Explored definitions and diagnostic stability of UM

Diagnostic baseline stability of UM over the follow-up time was calculated for each of the definitions not using follow-up data. This was done by calculating the percentage of UM cases not experiencing a depressive or mixed episode during follow-up. Cases meeting the UM definition due to an episode of mania during follow-up were not included in this calculation.

The original bipolar cohort included 315 individuals. Missing data and insufficient episodes to ascertain course of the disorder reduced the cohort size with ever increased strictness of definition. The six explored definitions were:

Definition 1 with 1 ≤ manic episodes at baseline resulted in a cohort with n = 289, unipolar manic course n = 179 (61.9%), bipolar course n = 110 (38.1 %), and a diagnostic stability of 78.3%.

Definition 2 with 1 ≤ manic episodes including follow-up data resulted in a cohort with n = 270, unipolar manic course n = 130 (48.1%), and bipolar course n = 140 (51.9 %).

Definition 3 with 2 ≤ manic episodes at baseline resulted in a cohort with n = 234, unipolar manic course n = 124 (53.0%), bipolar course n = 110 (47.0 %), and a diagnostic stability of 76.4%. 55 cases were not possible to define, having just one manic episode.

Definition 4 with 2 ≤ manic episodes including follow-up data resulted in a cohort with n = 240, unipolar manic course n = 100 (41.7%), and bipolar course n = 140 (58.3 %). 30 cases were not possible to define having just one manic episode. This definition was chosen for the subsequent study analysis.

Definition 5 with 3 ≤ manic episodes at baseline resulted in a cohort with n = 166, unipolar manic course n = 56 (33.7%), bipolar course n = 110 (66.3 %), and a diagnostic stability of 64.7%. 123 cases were not possible to define, having just one or two manic episodes.

Definition 6 with 3 ≤ manic episodes including follow-up data resulted in a cohort with n = 187, unipolar manic course n = 47 (25.1%), and bipolar course n = 140 (74.9 %). 83 cases were not possible to define, having just one or two manic episodes.

### Origins of secondary outcome data

Variables used in logistic regression:
*(numbers indicate SCAN item numbers)*
-"gender" (0.023),
-"Problems due to alcohol or other substances" as a proxy for "Khat use" (1.006, coded 0-3, "No evidence that symptom category was ever present.", "Present, but only mildly distressing or disabling.", "Present and moderately distressing or disabling.", Present and severely distressing or disabling."),
-“age of onset of disorder” (combining items for onset of depression and mania, 1.016 & 1.017)
 -social functioning (= transcoded SF-36 items 6 & 10) above population mean (Kebede et al. 2004).

## Results: IQRs (interquartile ranges)

Age in years: Median(IQR) = 28(22-33) for UM, and 30(23-36.5) for bipolar disorder.

Age of onset in years: Median(IQR) = 20 (IQR 17-25) years for UM, and 21.7 and 20 years (16-25) for bipolar disorder.

Duration of illness in years: Median(IQR) = 6 years for UM (2.5-11), and 7 years for bipolar disorder (3.5-14.5).

Social functioning: Median(IQR) = 62.5 for UM (50-87.5), and 50 for bipolar disorder (37.5-75).

**References:**

Andrews, G., and L. Peters. 1998. ‘The Psychometric Properties of the Composite International Diagnostic Interview’. *Soc Psychiatry Psychiatr Epidemiol* 33 (2): 80–88.

Brazier, J. E., R. Harper, N. M. Jones, A. O’Cathain, K. J. Thomas, T. Usherwood, and L. Westlake. 1992. ‘Validating the SF-36 Health Survey Questionnaire: New Outcome Measure for Primary Care’. *BMJ* 305 (6846): 160–64.

Byass, P., Y. Berhane, A. Emmelin, D. Kebede, T. Andersson, U. Hogberg, and S. Wall. 2002. ‘The Role of Demographic Surveillance Systems (DSS) in Assessing the Health of Communities: An Example from Rural Ethiopia’. *Public Health* 116 (3): 145–50. https://doi.org/10.1038/sj.ph.1900837.

E Rashid, D Kebede, and A Alem. 1996. ‘Evaluation of an Amharic Version of the CIDI and Prevalence Estimation of DSMIII-R Disorders in Addis Ababa.’ *Ethiop J Health Dev 1996;2:69–77.*

Fekadu, A. 2010. ‘Studies on Affective Disorders in Rural Ethiopia - Dissertation’.

Fekadu, A., D. Kebede, A. Alem, D. Fekadu, S. Mogga, A. Negash, G. Medhin, T. Beyero, and T. Shibre. 2006. ‘Clinical Outcome in Bipolar Disorder in a Community-Based Follow-up Study in Butajira, Ethiopia’. *Acta Psychiatrica Scandinavica* 114 (6): 426–34. https://doi.org/10.1111/j.1600-0447.2006.00825.x.

Hamilton, M. 1960. ‘A Rating Scale for Depression’. *J Neurol Neurosurg Psychiatry* 23 (February): 56–62.

Kebede, D., A. Alem, T. Shibire, N. Deyassa, A. Negash, T. Beyero, G. Medhin, and A. Fekadu. 2006. ‘Symptomatic and Functional Outcome of Bipolar Disorder in Butajira, Ethiopia’. *J Affect Disord* 90 (2–3): 239–49. https://doi.org/10.1016/j.jad.2005.11.009.

Kebede, D., A. Alem, T. Shibre, A. Negash, N. Deyassa, and T. Beyero. 2004. ‘Health Related Quality of Life (SF-36) Survey in Butajira, Rural Ethiopia: Normative Data and Evaluation of Reliability and Validity’. *Ethiop Med J* 42 (4): 289–97.

Kebede, D., A. Alem, T. Shibre, A. Negash, A. Fekadu, D. Fekadu, N. Deyassa, L. Jacobsson, and G. Kullgren. 2003. ‘Onset and Clinical Course of Schizophrenia in Butajira-Ethiopia--a Community-Based Study’. *Soc Psychiatry Psychiatr Epidemiol* 38 (11): 625–31. https://doi.org/10.1007/s00127-003-0678-4.

Leidy, N. K., C. Palmer, M. Murray, J. Robb, and D. A. Revicki. 1998. ‘Health-Related Quality of Life Assessment in Euthymic and Depressed Patients with Bipolar Disorder. Psychometric Performance of Four Self-Report Measures’. *J Affect Disord* 48 (2–3): 207–14.

Sartorius, N., and A. Janca. 1996. ‘Psychiatric Assessment Instruments Developed by the World Health Organization’. *Soc Psychiatry Psychiatr Epidemiol* 31 (2): 55–69.

Shibre, T., D. Kebede, A. Alem, A. Negash, S. Kibreab, A. Fekadu, D. Fekadu, L. Jacobsson, and G. Kullgren. 2002. ‘An Evaluation of Two Screening Methods to Identify Cases with Schizophrenia and Affective Disorders in a Community Survey in Rural Ethiopia’. *Int J Soc Psychiatry* 48 (3): 200–208.

Ware, J. E. 1993. ‘SF-36 Health Survey: Manual and Interpretation Guide. The Health Institute, New England Medical Centre, Boston’.

Ware, J. E., and C. D. Sherbourne. 1993. ‘The MOS 36-Item Short-Form Health Survey (SF-36). I. Conceptual Framework and Item Election. Med. Care 30, 473– 483.’

WHO. 1997. ‘Composite International Diagnostic Interview, Ver. 2.1 (CIDI 2.1). Geneva: WHO, 1997.’

Wing, J. K., T. Babor, T. Brugha, J. Burke, J. E. Cooper, R. Giel, A. Jablenski, D. Regier, and N. Sartorius. 1990. ‘SCAN. Schedules for Clinical Assessment in Neuropsychiatry’. *Arch Gen Psychiatry* 47 (6): 589–93.

Young, R. C., J. T. Biggs, V. E. Ziegler, and D. A. Meyer. 1978. ‘A Rating Scale for Mania: Reliability, Validity and Sensitivity’. *Br J Psychiatry* 133 (November): 429–35.
